# Chemical characterization of 21 species of marine macroalgae common in Norwegian waters: benefits of and limitations to their potential use in food and feed

**DOI:** 10.1002/jsfa.8798

**Published:** 2018-01-18

**Authors:** Irene Biancarosa, Ikram Belghit, Christian G Bruckner, Nina S Liland, Rune Waagbø, Heidi Amlund, Svenja Heesch, Erik‐Jan Lock

**Affiliations:** ^1^ National Institute of Nutrition and Seafood Research (NIFES) Bergen Norway; ^2^ Department of Biology University of Bergen Bergen Norway; ^3^ Norwegian Institute of Bioeconomy Research Bodø Norway; ^4^ Irish Seaweed Research Group, Ryan Institute National University of Ireland Galway Galway Ireland

**Keywords:** seaweeds, Norway, nutrients, arsenic, heavy metals

## Abstract

**BACKGROUND:**

In the past few years, much effort has been invested into developing a new blue economy based on harvesting, cultivating and processing marine macroalgae in Norway. Macroalgae have high potential for a wide range of applications, e.g. as source of pharmaceuticals, production of biofuels or as food and feed. However, data on the chemical composition of macroalgae from Norwegian waters are scant. This study was designed to characterize the chemical composition of 21 algal species. Both macro‐ and micronutrients were analysed. Concentrations of heavy metals and the metalloid arsenic in the algae were also quantified.

**RESULTS:**

The results confirm that marine macroalgae contain nutrients which are relevant for both human and animal nutrition, the concentrations whereof are highly dependent on species. Although heavy metals and arsenic were detected in the algae studied, concentrations were mostly below maximum allowed levels set by food and feed legislation in the EU.

**CONCLUSION:**

This study provides chemical data on a wide range of algal species covering the three taxonomic groups (brown, red and green algae) and discusses both benefits of and potential limitations to their use for food and feed purposes. © 2017 The Authors. Journal of The Science of Food and Agriculture published by John Wiley & Sons Ltd on behalf of Society of Chemical Industry.

## INTRODUCTION

Marine macroalgae or seaweeds are a large and heterogeneous group of photosynthetic organisms found worldwide in marine environments, commonly classified into three taxonomic groups: brown algae (Phaeophyceae), red algae (Rhodophyta) and green algae (Chlorophyta). Macroalgae are part of the traditional food culture of many Asian countries, where they have been cultivated on a large scale for centuries. In contrast to Asia, the exploitation of this resource in Europe has been very limited and mainly focused on the industrial production of thickeners (e.g. agar and alginates).^1^ Several algal species can be found growing naturally in enormous volumes along the coastline of Norway, which is among the world's longest and most productive, enhancing the interest to utilize this resource both wild‐harvested and cultivated.^2^ In the last decade, in Norway, an increasing number of research projects have focused on the use of algae for a wide range of applications,^2^ such as industrial production of biofuels^3^ and compounds of medical and pharmaceutical value.^4^ Algae are naturally rich in valuable nutrients such as minerals, vitamins and polyunsaturated fatty acids (PUFAs) (e.g. eicosapentaenoic acid (EPA)).^5^ Moreover, certain species common in Norwegian waters can contain relatively high protein levels (200–300 g kg^−1^ dry weight (DW)) and a considerable amount of essential amino acids.^6^, ^7^ These properties, coupled with high variations in shape, colour, texture and taste, make marine macroalgae attractive as food and feed items.^2^ In recent decades, there has been increasing interest in eating macroalgae in Norway, with the most relevant food species being the brown algae *Saccharina latissima* (sugar kelp) and *Alaria esculenta* (winged kelp) and the red algae *Porphyra* sp. (red and purple laver) and *Palmaria palmata* (red sea lettuce).^8^ Moreover, marine macroalgae have also seen renewed interest as feed ingredients for livestock (e.g. ruminants, pigs and poultry), especially the species *Ascophyllum nodosum* (rockweed) and *Laminaria* sp. (kelp).^9^

Marine macroalgae can contain high concentrations of iodine.^10^ Iodine is a trace element essential for the synthesis of the thyroid hormones thyroxine (T3) and triiodothyronine (T4) involved in the regulation of metabolism in both humans and animals. An insufficient dietary supply of this element can lead to the development of several disorders such as thyroid function abnormalities, goitre and cretinism, whereas excess intake has been shown to cause toxic effects in humans and fish.^11^, ^12^ Marine macroalgae can accumulate undesirable elements from the surrounding environment, especially certain metals and arsenic (As) in high concentrations,^13^, ^14^ which can be toxic to living organisms.^15^ Documentation of both nutrients and undesirable elements potentially present in algae is fundamental to determine potentials and limitations of their use for food and feed purposes. However, such data on species from Norwegian waters^6^, ^7^, ^16^ are very scarce.

In the present study, we characterized the chemical composition of 21 species of marine macroalgae collected along the Norwegian coast, representing the three groups of red, green and brown algae. We also determined concentrations of the heavy metals cadmium (Cd), lead (Pb) and mercury (Hg) and the metalloid As. We discuss differences among the species studied, assessing benefits of and limitations to their potential use as food and feed ingredients.

## MATERIALS AND METHODS

### Sample collection and species identification

Macroalgae were harvested in October 2014 along the Northern coast of Norway (between 67.24 and 67.32° N and 14.47 and 14.72° E) in the intertidal or upper subtidal zone. Each sample consisted of pooled material of at least five individuals per species. The processing of the samples is described in detail in Biancarosa *et al*.^7^ Briefly, the algae were rinsed in cold freshwater to remove adhering foreign material, then ground, powdered and stored at −30 °C prior to analyses.

A complete list of the species identified in the current study as well as the sample locations is given in Table 1.

**Table 1:** Marine macroalgal species included in study^a^ and coordinates of sampling locations

No.	Taxon	Species	Common name^b^	GPS coordinates
1	Rhodophyta (red algae)	*Porphyra dioica* J. Brodie & L.M. Irvine	Black laver	67.323491, 14.478753
2		*Porphyra purpurea* (Roth) Agardh	Purple laver	67.323491, 14.478753
3		*Porphyra umbilicalis* Kützing	Tough laver	67.239783, 14.510323
4		*Chondrus crispus* Stackhouse	Irish moss	67.412274, 14.621368
5		*Mastocarpus stellatus* (Stackhouse) Guiry	Grape pip weed	67.325565, 14.478626
6		*Furcellaria lumbricalis* (Hudson) J.V. Lamouroux	Clawed fork weed	67.305987, 14.727638
7		*Palmaria palmata* (L.) Weber & Mohr	Dulse	67.322567, 14.457314
8	Chlorophyta (green algae)	*Ulva intestinalis* L.	Gut weed	67.323491, 14.478753
9		*Ulva lactuca* L.	Sea lettuce	67.323491, 14.478753
10		*Cladophora rupestris* (L.) Kützing	Common green branched weed	67.305987, 14.727638
11	Phaeophyceae (brown algae)	*Fucus serratus* L.	Serrated wrack	67.323491, 14.478753
12		*Fucus vesiculosus* L.	Bladder wrack	67.240804, 14.712079
13		*Fucus spiralis* L.	Spiral wrack	67.305987, 14.727638
14		*Pelvetia canaliculata* (L.) Decaisne & Thuret	Channel wrack	67.326911, 14.478223
15		*Halidrys siliquosa* (L.) Lyngbye	Sea oak	67.239783, 14.510323
16		*Himanthalia elongata* (L.) S.F. Gray	Thong weed	67.276063, 14.572370
17		*Ascophyllum nodosum* (L.) Le Jolis	Egg wrack	67.305987, 14.727638
18		*Saccharina latissima* (L.) C.E. Lane, C. Mayes, Druehl & G.W. Saunders	Sugar tang	67.240804, 14.712079
19		*Laminaria digitata* (Hudson) J.V. Lamouroux	Sea girdle	67.240804, 14.712079
20		*Alaria esculenta* (L.) Greville	Wing kelp	67.276063, 14.572370
21		*Chordaria flagelliformis* (O.F. Müller) C. Agardh	Slimy whip weed	67.239783, 14.510323

a See Biancarosa *et al*.^7^ for European Nucleotide Archive (ENA)/GenBank accession numbers.

b According to http://www.algaebase.org.

### Chemical analyses

Dry matter (DM) content was estimated gravimetrically by freeze‐drying the samples at −20 °C in vacuum (0.2–0.01 mbar) for 24 h and then leaving them in vacuum at 25 °C until constant weight was reached.

Fatty acid (FA) composition was quantified by gas chromatography coupled with flame ionisation detection using a method described by Torstensen *et al*.^17^

Multi‐element analysis was carried out by inductively coupled plasma mass spectrometry (ICPMS) after wet digestion in a microwave oven, similarly to Julshamn *et al*.^18^

Inorganic As (iAs) was analysed by anion exchange high‐pressure liquid chromatography coupled with ICPMS (HPLC/ICPMS), based on Sloth *et al*.^19^

Iodine was quantified according to Julshamn *et al*.^18^ using ICPMS (Agilent 7500, Agilent, Santa Clara, CA, USA) coupled with autosampling (ASX‐500, Cetac, Omaha, NE, USA).

Vitamin E forms were analysed by HPLC according to Konings *et al*.^20^ as described by Hamre *et al*.^21^

## RESULTS

### Fatty acid profile

Concentrations of palmitoleic acid (16:1*n*‐7), hexadecatrienoic acid (16:3*n*‐3), vaccenic acid (18:1*n*‐7) and *α*‐linolenic acid (18:3*n*‐3) in green algal species were 0.03–0.98, 0.01–0.18, 0.20–0.46 and 0.11–0.97 mg g^−1^ DW of total FAs respectively; while in red and brown algal samples these FAs reached concentrations of 0.01–046, <LOQ (below limit of quantification)–0.3, 0.01–0.16 and <LOQ–1.51 mg g^−1^ DW respectively (Table 2). Total saturated fatty acid (SFAs) amounted to 0.96–1.7, 1.23–9 and 0.04–2.3 mg g^−1^ DW in green, brown and red algae respectively (Table 2). Palmitic acid (16:0) was the most abundant SFA in all algal samples. Concentrations of monounsaturated fatty acids (MUFAs) were highest in brown algae (0.64–21 mg g^−1^ DW), comprising mainly oleic acid (18:1*n*‐9) (0.48–20 mg g^−1^ DW). Concentrations of PUFAs were 2.1–2.3, 2–19 and 0.03–5 mg g^−1^ DW in green, brown and red algae respectively.

**Table 2:** Fatty acid composition (mg g^−1^ algal DW) of 21 macroalgal species

Species	14:0	16:0	18:0	Sum SFAs	16:1*n*‐7	18:1*n*‐9	18:1*n*‐7	Sum MUFAs	16:3*n*‐3	18:2*n*‐6 (LA)	18:3*n*‐3 (ALA)	20:4*n*‐6	20:5*n*‐3 (EPA)	Sum PUFAs	Sum *n*‐3	Sum *n*‐6	*n*‐6/*n*‐3
Red algae																	
*C. crispus*	0.01	0.04	0.01	0.04	0.01	0.02	0.01	0.02	0.3	<LOQ	<LOQ	0.01	0.01	0.03	0.01	0.01	0.9
*F. lumbricalis*	0.06	0.65	0.02	0.74	0.14	0.25	0.02	0.41	<LOQ	0.02	0.01	0.36	0.92	1.34	0.92	0.40	2.3
*M. stellatus*	0.08	0.62	0.03	0.75	0.08	0.42	0.05	0.58	0.01	0.04	0.03	0.72	0.57	1.45	0.63	0.80	0.8
*P. palmata*	0.23	0.74	0.03	1.04	0.03	0.16	0.08	0.32	0.02	0.14	0.20	0.13	1.50	2.31	2.00	0.31	6.4
*P. dioica*	0.06	2.03	0.11	2.31	0.11	0.28	0.16	0.74	<LOQ	0.23	0.10	1.06	2.79	4.76	3.11	1.64	1.9
*P. purpurea*	0.02	0.47	0.02	0.51	0.02	0.08	0.08	0.32	<LOQ	0.06	0.01	0.13	0.86	1.17	0.88	0.28	3.1
*P. umbilicalis*	0.06	0.39	0.03	0.50	0.06	0.11	0.05	0.27	0.2	0.06	0.08	0.13	0.70	1.25	0.96	0.27	3.5
Green algae																	
*C. rupestris*	0.41	1.12	0.04	1.66	0.98	0.28	0.20	1.50	0.01	1.12	0.11	0.16	0.32	2.33	0.80	1.34	0.6
*U. intestinalis*	0.03	0.86	0.02	0.95	0.05	0.03	0.46	0.56	0.18	0.29	0.97	0.02	0.05	2.17	1.80	0.36	4.9
*U. lactuca*	0.03	1.05	0.04	1.19	0.03	0.10	0.43	0.58	0.13	0.27	0.78	0.08	0.10	2.13	1.68	0.43	3.9
Brown algae																	
*A. esculenta*	0.28	0.98	0.08	1.43	0.13	0.99	0.03	1.15	<LOQ	0.38	0.25	0.74	0.48	2.31	1.09	1.21	0.9
*A. nodosum*	1.83	2.05	0.12	4.16	0.29	8.62	0.04	9.22	<LOQ	1.78	0.49	2.50	1.09	7.23	2.08	5.12	0.4
*C. flagelliformis*	1.58	2.88	0.77	5.59	0.04	3.54	<LOQ	3.58	<LOQ	2.86	0.99	1.23	1.76	8.75	4.34	4.41	1.0
*F. serratus*	2.65	3.23	0.14	6.23	0.30	10.31	0.03	10.9	<LOQ	2.52	0.71	2.52	0.95	7.67	1.99	5.63	0.4
*F. spiralis*	4.65	3.63	0.33	9.05	0.46	19.69	0.04	20.9	<LOQ	4.28	1.45	3.91	1.57	13.5	3.89	9.57	0.4
*F. vesiculosus*	2.82	2.40	0.14	5.60	0.24	8.09	0.02	8.61	<LOQ	2.83	1.09	3.02	1.30	9.81	3.07	6.71	0.5
*H. siliquosa*	0.39	1.24	0.07	1.88	0.05	1.12	0.01	1.21	<LOQ	0.38	0.45	1.10	0.42	2.96	1.37	1.57	0.9
*H. elongata*	0.26	1.17	0.03	1.57	0.10	0.62	0.01	0.76	<LOQ	0.44	0.43	0.91	0.46	2.58	1.14	1.43	0.8
*L. digitata*	0.29	1.09	0.05	1.56	0.13	1.23	0.01	1.41	<LOQ	0.56	0.42	0.59	0.82	3.10	1.89	1.21	1.6
*P. canaliculata*	2.50	2.70	0.76	6.51	0.45	17.37	0.04	18.2	<LOQ	4.99	1.51	6.32	2.06	18.8	4.57	14.1	0.3
*S. latissima*	0.45	0.67	0.04	1.23	0.13	0.48	0.01	0.64	<LOQ	0.33	0.24	0.48	0.39	2.03	1.15	0.88	1.3

Data represent mean values of two analytical measurements conducted on pooled algal material of several individuals per species. SFAs, saturated fatty acids; MUFAs, monounsaturated fatty acids; LA, linoleic acid; ALA, *α*‐linolenic acid; EPA, eicosapentaenoic acid; PUFAs, polyunsaturated fatty acids; LOQ, limit of quantification (0.1 area %).

### Elemental composition

A detailed overview of the mineral composition of the algae in this study is presented in Table 3. Iodine contents of the algae ranged from <200 mg kg^−1^ DW in most red algal species to >3000 mg kg^−1^ DW in some brown algal species such as *S. latissima* (4600 mg kg^−1^ DW) and *Laminaria digitata* (10 000 mg kg^−1^ DW).

**Table 3:** Macro‐ and micromineral concentrations (g kg^−1^ algal DW for Ca, Mg, P, K and Na; mg kg^−1^ algal DW for Cu, Fe, I, Mn, Se and Zn) of 21 macroalgal species

Species	Ca	Mg	P	K	Na	Cu	Fe	I	Mn	Se	Zn
Red algae											
*C. crispus*	13	9	2.4	30	18	7.6	330	200	22	0.14	55
*F. lumbricalis*	3.7	8.9	1.2	42	10	6.2	130	84	7.5	0.1	23
*M. stellatus*	6.7	7.9	1.4	20	27	3.7	200	340	7.1	0.1	72
*P. palmata*	2.5	1.2	2.1	28	3.2	4.1	73	220	4.1	0.1	42
*P. dioica*	19	3.8	3.3	26	4	10	570	84	25	0.29	24
*P. purpurea*	5.4	17	3.3	31	100	8.0	89	22	6.7	0.05	29
*P. umbilicalis*	7	3.8	2.5	17	4.4	8.8	160	110	21	0.17	67
Green algae											
*C. rupestris*	8.6	4.0	1.6	21	1.8	7.0	930	480	56	0.68	13
*U. intestinalis*	29	11	1.7	12	8.5	5.7	5800	130	180	0.76	21
*U. lactuca*	16	27	2.2	28	7.0	7.1	1800	43	26	0.14	19
Brown algae											
*A. esculenta*	22	7.9	3.7	54	16	2.0	72	380	3.7	0.18	55
*A. nodosum*	17	8.6	0.83	17	33	3.6	100	670	13	0.06	84
*C. flagelliformis*	16	8.2	2.3	34	21	1.0	63	1100	140	0.12	43
*F. serratus*	16	7.4	0.76	30	32	2.1	240	440	69	0.09	37
*F. spiralis*	17	8.2	1.1	28	27	2.5	120	150	33	0.09	42
*F. vesiculosus*	30	6.7	1.0	25	18	3.7	290	260	37	0.08	28
*H. siliquosa*	16	6.2	1.1	36	13	0.77	16	710	2.7	0.03	16
*H. elongata*	18	9.4	1.5	47	39	1.1	20	59	6.1	0.05	23
*L. digitata*	15	6.3	1.6	31	27	1.3	150	10000	3.1	0.07	81
*P. canaliculata*	14	7.9	0.70	17	23	3.9	300	200	8.0	0.05	28
*S. latissima*	17	7.7	2.5	100	24	1.2	160	4600	5.7	0.06	25

Data represent mean values of two analytical measurements conducted on pooled algal material of several individuals per species. Ca, calcium; Mg, magnesium; P, phosphorus; K, potassium; Na, sodium; Cu, copper; Fe, iron; I, iodine; Mn, manganese; Se, selenium; Zn, zinc.

**Figure 1:**
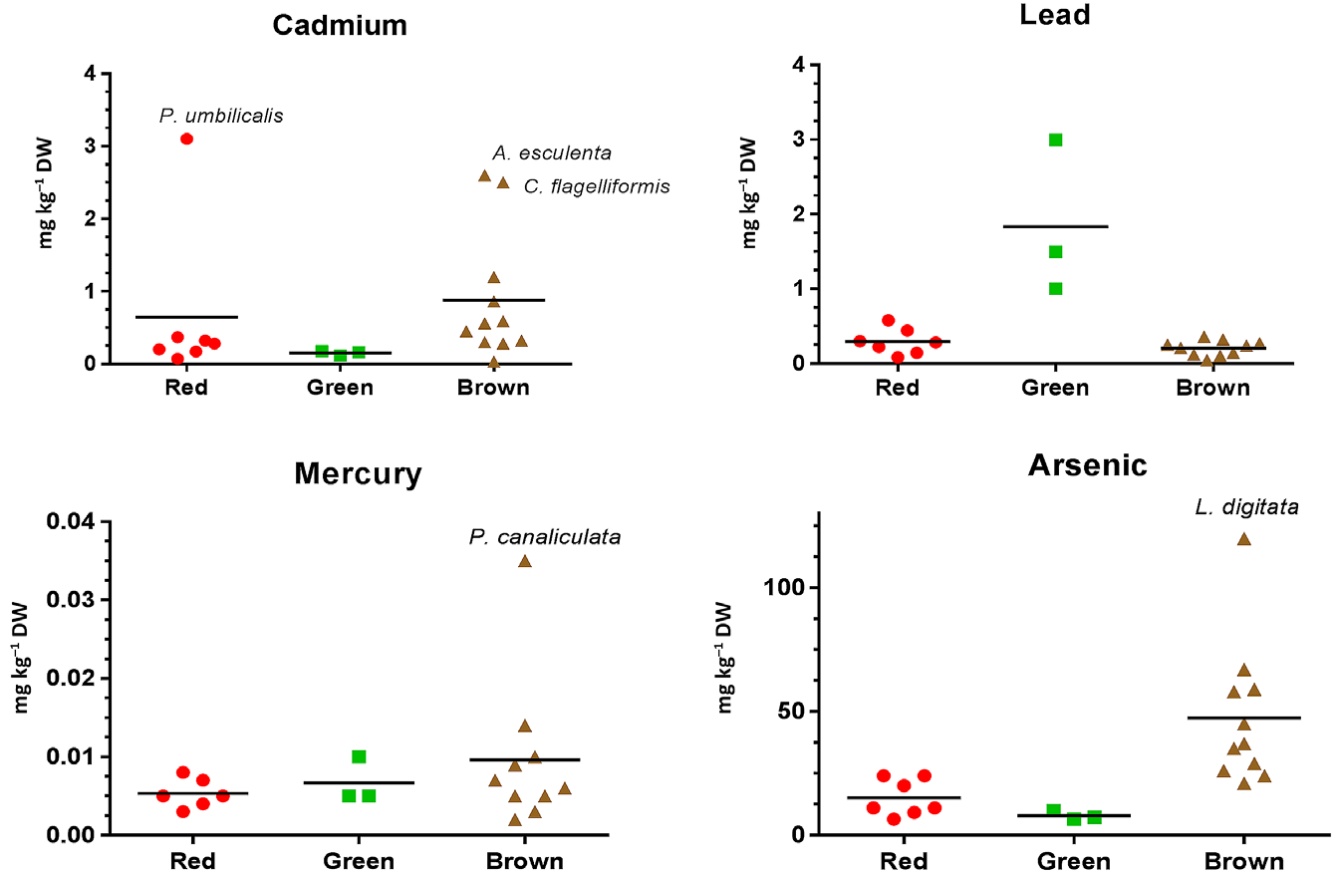
Concentrations of heavy metals cadmium, lead and mercury and metalloid arsenic (total) in red, green and brown algae. Horizontal lines indicate average values.

The heavy metals Cd, Hg and Pb were found in all samples analysed, with their concentrations varying widely between species (Fig. 1; Supplementary Table 1). The level of Cd was relatively low in green algae (0.12–0.18 mg kg^−1^ DW) compared with red and brown algae (0.07–3.1 and 0.03–2.6 mg kg^−1^ DW respectively). The concentrations of Hg in the species studied ranged from <LOQ to 0.04 mg kg^−1^ DW (*Pelvetia canaliculata*). In this study, Pb was found to be low in red and brown algae (up to 0.58 mg kg^−1^ DW in *Porphyra dioica*) compared with green algae (up to 3 mg kg^−1^ DW in *Ulva intestinalis*).

Arsenic content (as total As) in the samples is shown in Fig. 1. Higher levels of this metalloid were found in brown algae (21–120 mg kg^−1^ DW) compared with red (6.4–24 mg kg^−1^ DW) and green (6.4–10 mg kg^−1^ DW) algae. Levels of inorganic As (iAs) were generally low in the species studied (mostly below 0.5 mg kg^−1^ DW) (Supplementary Table 1). However, in the brown alga *Halidrys siliquosa* the concentration of iAs was 2.4 mg kg^−1^ DW, amounting to 10% of total As.

### Vitamin E

Brown algae had higher contents of *α*‐, *β*‐, *γ*‐ and *δ*‐tocopherol (6.2–93, 0.06–23, 0.07–179 and <LOQ–194 mg kg^−1^ DM respectively) compared with red and green algae, which contained only low levels of *α*‐tocopherol (10–26 and 8.8–12 mg kg^−1^ DM respectively). Tocotrienol was not detected or <1 mg kg^−1^ DW in all samples except for the brown alga *H. siliquosa* (*α*‐, *β*‐ and *γ*‐tocotrienol: 3.8, 8.7 and 3.2 mg kg^−1^ DW respectively) (Table 4).

**Table 4:** Vitamin E composition (mg kg^−1^ algal DW) of 21 macroalgal species

Species	*α*‐Tocopherol	*β*‐Tocopherol	*γ*‐Tocopherol	*δ*‐Tocopherol	*α*‐Tocotrienol	*β*‐Tocotrienol	*γ*‐Tocotrienol	*δ*‐Tocotrienol
Red algae								
*C. crispus*	9.6	0.10	0.06	<LOQ	<LOQ	0.21	<LOQ	<LOQ
*F. lumbricalis*	14.4	0.05	0.05	<LOQ	<LOQ	0.18	<LOQ	<LOQ
*M. stellatus*	16.0	<LOQ	0.07	<LOQ	<LOQ	0.30	<LOQ	<LOQ
*P. palmata*	13.3	0.04	0.32	0.06	<LOQ	0.76	<LOQ	<LOQ
*P. dioica*	26	0.24	0.25	<LOQ	<LOQ	0.18	<LOQ	<LOQ
*P. purpurea*	10.1	0.05	0.16	0.09	<LOQ	0.19	<LOQ	<LOQ
*P. umbilicalis*	13.1	0.16	0.23	0.21	<LOQ	<LOQ	<LOQ	<LOQ
Green algae								
*C. rupestris*	12.0	<LOQ	0.10	0.07	<LOQ	<LOQ	<LOQ	<LOQ
*U. intestinalis*	8.8	<LOQ	0.11	<LOQ	<LOQ	0.11	<LOQ	<LOQ
*U. lactuca*	NA	NA	NA	NA	NA	NA	NA	NA
Brown algae								
*A. esculenta*	24	0.18	0.75	0.11	<LOQ	0.15	LOQ	<LOQ
*A. nodosum*	80	8.1	51	194	0.29	0.52	0.62	<LOQ
*C. flagelliformis*	51	0.18	1.0	LOQ	<LOQ	0.90	<LOQ	<LOQ
*F. serratus*	44	10.3	15.3	82	0.20	<LOQ	0.58	0.46
*F. spiralis*	68	23	12.9	144	0.13	0.88	0.14	LOQ
*F. vesiculosus*	60	14	9.3	94	0.16	0.33	0.10	LOQ
*H. siliquosa*	67	1.8	179	30	3.8	8.7	3.2	0.82
*H. elongata*	65	0.29	5.0	0.26	<LOQ	0.13	<LOQ	<LOQ
*L. digitata*	6.2	0.06	0.07	<LOQ	<LOQ	1.06	<LOQ	<LOQ
*P. canaliculata*	93	18	20	123	0.36	1.2	0.10	0.15
*S. latissima*	13	0.16	0.10	0.83	<LOQ	<LOQ	<LOQ	<LOQ

Data represent mean values of two analytical measurements conducted on pooled algal material of several individuals per species. NA, not analysed; LOQ, limit of quantification (0.08 mg kg^−1^ DW).

## DISCUSSION

The macroalgal samples collected in this study contain nutrients such as omega‐3 fatty acids, iodine and vitamin E which can be relevant for food and feed purposes; however, they also contain undesirable elements such as Cd and As. Here we will discuss benefits and potential limitations to the use of the species studied for food and feed purposes.

### Fatty acid profile

The FA compositions of the algae studied varied not only between the three phyla but also between different species belonging to the same phylum. This is consistent with previous reports and allows the FA profiles to be used for chemotaxonomic analysis to differentiate taxonomic groups.^22^ The FA profiles of green algae differed from those of brown and red algae and showed more resemblance to the FA profiles of related terrestrial plants. In accordance with previous studies, the green algae in the current study contained higher concentrations of C16 and C18 PUFAs such as linoleic acid (18:2*n‐*6) and *α*‐linolenic acid (18:3*n*‐3).^23^, ^24^

In red algae, high relative concentrations of EPA (36% of total FAs) were observed, especially in *Porphyra* species, where this marine omega‐3 fatty acid comprised more than a third of total FAs. High relative concentrations of this long‐chain (LC) PUFA have previously been reported in red algal species.^6^, ^25^ EPA is well known for its beneficial effects on health, especially against cardiovascular diseases.^26^ On the other hand, another health‐promoting marine omega‐3 PUFA, docosahexaenoic acid (DHA), was not present in the samples analysed in this study, confirming previous findings.^6^, ^25^ Despite their high concentrations of EPA, red algae cannot be considered good dietary sources of LC *n*‐3 PUFAs owing to their low total lipid contents. Currently, the global recommendation for intake of EPA + DHA is about 200–250 mg day^−1^,^15^ and while the use of red algae as stand‐alone oil‐based dietary products is thus unlikely compared with other sources of marine omega‐3 PUFAs such as microalgae or fish,^27^, ^28^ they may still be used as supplements in diets for both human and animal nutrition.

An imbalance between *n*‐6 and *n*‐3 FAs in biological tissues is known to cause inflammatory processes in the body.^29^ Thus the ratio between *n*‐6 and *n*‐3 FAs is considered an index for evaluating the nutritional value of a dietary lipid source with respect to human and animal development and health.^29^ Today, the ratio between *n*‐6 and *n*‐3 is around 15–20:1 in Western diets^30^; this contrasts with the ideal ratio, which should not exceed <5:1, as recommended by the World Health Organization (WHO).^31^ Since the *n*‐6/*n*‐3 ratio of the algae in this study was within the recommended range of <5:1, they have the potential to enhance the nutritional quality of food products, e.g. by regulating low‐density lipoprotein and cholesterol levels, and thus may help to prevent inflammatory, cardiovascular diseases and nervous system disorders. Likewise, macroalgae with low *n*‐6/*n*‐3 ratio and high *n*‐3 LC PUFA contents could improve the FA composition of farmed fish species.^32^

### Elements

The species in this study were found to contain macro‐ and microminerals which are relevant for both feed and food purposes. For example, calcium in *P. dioica*, *U. intestinalis* and *Fucus vesiculosus* reached 19, 29 and 30 g kg^−1^ algal DW respectively. This indicates that eating a 10 g portion of these dry macroalgae provides approximately 24, 36 and 37% respectively of the recommended daily intake of calcium for adult males and females in Nordic countries.^33^ Moreover, at 5800 mg kg^−1^ DW, the level of iron in the green alga *U. intestinalis* is higher than in many well‐known terrestrial sources of this mineral such as leafy green vegetables, legumes, nuts and cereal grains, which all contain between 2 and 4 mg iron per 100 g.^34^ Thus exploring this marine macroalga as a natural food resource could be a solution to prevent iron deficiency, which is one of the most prevalent nutritional deficiencies in the word.^31^ Major sources of iodine in Norwegian foods are seafood, milk and dairy products, with lean fish species such as cod (*Gadus morhua*) having among the highest iodine contents (86 μg kg^−1^ wet weight (WW) on average).^35^ In this study, the iodine contents of the algae were generally high, ranging from 22 to 10 000 mg kg^−1^ DW, although variability among different species and phylogenetic groups (red, green and brown algae) was considerable. The uptake of iodine in algae has been shown to be dependent on several factors such as salinity and temperature of the surrounding water, depth, and age of the thalli. The iodine values found in this study are in accordance with previous data on macroalgae collected in Norway and worldwide^6^, ^16^ and confirm that Norwegian marine macroalgae are good sources of iodine. Among the three taxonomic groups, brown algae can accumulate iodine in high concentrations.^6^ In the current study, very high iodine contents were found in the brown alga *Chordaria flagelliformis* (1100 mg kg − 1 DW) and the kelps *S. latissima* (4600 mg kg^−1^ DW) and *L. digitata* (10 000 mg kg^−1^ DW). Since excess iodine can cause adverse health effects such as dysfunctions of the thyroid gland, dietary uptake of these algae may have to be limited.

### Heavy metals and arsenic

The heavy metals Cd, Hg and Pb were found in all species studied. Accumulation of these undesirable elements, naturally present in marine environments, can easily occur in marine organisms, including macroalgae.^13^ The level of Cd was relatively low in green algae compared with red and brown algae. Previous data on macroalgae collected in Norway support these findings, as lower levels of this metal were found in green algae than in the other taxonomic groups.^6^, ^16^

The concentrations of Hg were relatively low in all species studied, in line with previous findings.^6^, ^14^, ^16^ Lead was found to be low in red and brown algae, while its concentrations in green algae were higher, especially in the green alga *U. intestinalis* (up to 3 mg kg^−1^ DW). Interestingly, Duinker *et al*.^16^ reported low levels of Pb in the green alga *Ulva lactuca* (0.18–0.23 mg kg^−1^ DW) collected in the south of Norway during spring/summer. Variability of metal levels in algae can be high among different species, seasons and collection sites.^6^, ^36^, ^37^ Moreover, a seasonal pattern in metal accumulation has been found in *Ulva* sp., with lowest metal concentrations in spring/summer and highest in autumn/winter.^38^ For Pb and Hg, EU legislation sets maximum levels for these elements in food supplements (which also apply for macroalgae) at 3 and 0.1 mg kg^−1^ WW respectively. These levels were not exceeded by any of the Pb and Hg concentrations found in the algae in this study (up to 0.3 and 0.01 mg kg^−1^ WW for Pb and Hg respectively).

Arsenic in biological matrices exists either in organic forms (e.g. arsenobetaine and arsenosugars) or as iAs.^39^ While organoarsenic forms are considered to be non‐toxic or of low toxicity, iAs is regarded as the most toxic form of As for living organisms.^15^ In the current study, As content in the samples was quantified as total As and iAs. Overall, higher levels of total As were found in brown algae than in the other taxonomic groups. This gradation of total As in relation to the group of algae (brown > red > green) has been previously shown in studies conducted on macroalgae from Norwegian waters^6^, ^16^, ^40^ as well as on macroalgae collected worldwide.^14^, ^41^, ^42^

Levels of iAs in the species studied comprised overall <7% of total As; that is, As present in these macroalgae was found to be mainly in organic forms. Previous studies on As speciation have shown that the most abundant form of this metalloid in macroalgae is organic.^40^, ^43^ However, in the current study, some species of brown algae had high levels of iAs, e.g. the brown alga *H. siliquosa* in which the concentration of iAs (2.4 mg kg^−1^ DW) reached 10% of total As. Data on concentrations of iAs in macroalgal species from Norwegian waters are scarce^16^; however, previous studies revealed that iAs levels in some brown algae reached between 20 and 80% of total As.^14^, ^40^, ^41^ The presence of As in macroalgae has safety implications for their use as food or feed. However, regulations on As in food are currently limited in the EU, and no maximum allowed levels of As (either total As or iAs) in vegetables or food supplements exist.^44^ The presence of As in feed in the EU is regulated by Directive 2002/32/EC^45^ and amendments, which set the maximum allowed level of this metalloid at 40 mg kg^−1^ (120 g kg^−1^ moisture content) for ‘seaweed meal and feed materials derived from seaweed’. This maximum level is set for total As, but authorities can request documentation showing that concentrations of iAs in feed materials are below 2 mg kg^−1^ (120 g kg^−1^ moisture content). In the current study, all species of red and green algae contained As concentrations below the EU current allowed level of 40 mg kg^−1^. However, four species of brown algae had levels of total As exceeding the maximum level allowed for algal feed materials, thus limiting the use of these algal species as feed ingredients in the EU.

### Vitamin E

Brown algae had high contents of *α*‐, *β*‐, *γ*‐ and *δ*‐tocopherol, while red and green algae contained only low levels of *α*‐tocopherol. The abundance of tocopherols detected in the present work was in accordance with earlier reports where brown algae were shown to contain higher levels of tocopherols than green and red algae.^46^ Among brown algae, the contents of the four forms of tocopherol vary between close relatives within a single genus. For example, the contents of *α*‐, *β*‐, *γ*‐ and *δ*‐tocopherol range from a low level in the order Laminariales (*A. esculenta*: 24, 0.18, 0.75 and 0.11 mg kg^−1^ DM respectively) to a high level in the order Fucales (*A. nodosum*: 80, 8.1. 51 and 194 mg kg^−1^ DM respectively). The higher abundance of tocopherols in these species corroborates previous studies where the Fucales order seems to be unique regarding tocopherol composition.^47^

## CONCLUSIONS

Based on our combined results on beneficial compounds and undesirable substances in the algal samples, red and green algae are the most promising algal groups for utilization in food and feed, especially *Poprhyra* and *Ulva* species. These macroalgae could serve as good sources of high‐quality lipids and minerals. However, animal trials using seaweeds are needed to assess the bioavailability of these nutrients. Some of the brown algae in this study (e.g. *L. digitata*, *S. latissima* and *A. esculenta*) contain high levels of arsenic and iodine, which could hamper their utilization for food and feed purposes. More data on seasonal and geographical variability are needed in order to assess the suitability of marine macroalgae collected in Norwegian waters for their use in food and feed.

## Supporting information


**Supplementary Table 1**. **Heavy metals and arsenic composition of 21 macroalgal species.** Data represent mean values of two analytical measurements conducted on pooled algal material of several individuals per species. Data are expressed as mg/kg of the algal dry weight (dw). Values in brackets refer to concentrations of inorganic arsenic expressed as mg/kg of the algal DW.
