# Supplementary material for: Chemical characterization of 21 species of marine macroalgae common in Norwegian waters: benefits of and limitations to their potential use in food and feed
Source: J Sci Food Agric. 2018 Jan 18;98(5):2035–42. doi: 10.1002/jsfa.8798 (PMC5888138; doi:10.1002/jsfa.8798)
Supplement: Supplementary file 1 — Supplementary Table 1. Heavy metals and arsenic composition of 21 macroalgal species. Data represent mean values of two analytical measurements conducted on pooled algal material of several individuals per species. Data are expressed as mg/kg of the algal dry weight (dw). Values in brackets refer to concentrations of inorganic arsenic expressed as mg/kg of the algal DW. [file JSFA-98-2035-s001.docx]

S**upplementary Table 1. Heavy metals and arsenic composition of 21 macroalgal species.** Data represent mean values of two analytical measurements conducted on pooled algal material of several individuals per species. Data are expressed as mg/kg of the algal dry weight (dw). Values in brackets refer to concentrations of inorganic arsenic expressed as mg/kg of the algal DW.

| Species | Cd | Pb | Hg | As |
| --- | --- | --- | --- | --- |
| Red algae |  |  |  |  |
| *C. crispus* | 0.28 | 0.3 | 0.005 | 24 (0.21) |
| *F. lumbricalis* | 0.07 | 0.22 | 0.004 | 6.4 (0.07) |
| *M. stellatus* | 0.2 | 0.28 | <LOQ | 11 (0.06) |
| *P. palmata* | 0.37 | 0.14 | 0.003 | 9.2 (0.02) |
| *P. dioica* | 0.32 | 0.58 | 0.008 | 24 (0.24) |
| *P. purpurea* | 0.17 | 0.44 | 0.005 | 11 (0.04) |
| *P. umbilicalis* | 3.1 | 0.08 | 0.007 | 20 (0.04) |
| Green algae |  |  |  |  |
| *C. rupestris* | 0.16 | 1.5 | 0.01 | 10 (0.25) |
| *U. intestinalis* | 0.18 | 3.0 | 0.005 | 6.4 (0.44) |
| *U. lactuca* | 0.12 | 1.0 | 0.005 | 7.2 (0.389 |
| Brown algae |  |  |  |  |
| *A. esculenta* | 2.5 | 0.14 | 0.005 | 59 (0.05) |
| *A. nodosum* | 0.32 | 0.10 | 0.014 | 35 (0.09) |
| *C. flagelliformis* | 2.6 | 0.36 | 0.002 | 26 (0.24) |
| *F. serratus* | 1.2 | 0.32 | 0.003 | 67 (0.03) |
| *F. spiralis* | 0.45 | 0.27 | 0.005 | 21 (0.03) |
| *F. vesiculosus* | 0.86 | 0.25 | 0.007 | 45 (0.11) |
| *H. siliquosa* | 0.28 | <LOQ | 0.006 | 24 (2.4) |
| *H. elongata* | 0.56 | 0.046 | <LOQ | 37 (0.04) |
| *L. digitata* | 0.033 | 0.12 | 0.009 | 120 (1.1) |
| *P. canaliculata* | 0.30 | 0.24 | 0.035 | 29 (0.09) |
| *S. latissima* | 0.59 | 0.21 | 0.010 | 58 (0.25) |

Abbreviations: Cd = cadmium; Pb = lead; Hg = mercury; As = arsenic; LOQ = limit of quantification (0.005 mg/kg for Cd and Hg, 0.03 mg/kg for Pb, 0.01 mg/kg for As).
